# Online-Based Recruitment Methods for Community-Dwelling Older Adults: Scoping Review and Lessons Learned From the PLAN Trial

**DOI:** 10.2196/55082

**Published:** 2025-02-25

**Authors:** Deborah Min, Ji-Young Yun, Chad Parslow, Anushka Jajodia, Hae-Ra Han

**Affiliations:** 1 Bloomberg School of Public Health Johns Hopkins University Baltimore, MD United States; 2 Grossman School of Medicine New York University New York, NY United States; 3 School of Nursing Johns Hopkins University Baltimore, MD United States

**Keywords:** older adults, online, online recruitment, community-dwelling, strategies, America, Americans, technology adoption, digital technologies, COVID-19, pandemic, digital health, dementia, caregivers, healthcare system, community health workers, consultants, mobile phone

## Abstract

**Background:**

Despite rapid technological advancement and a considerably aging US population, there remains a gap in the literature pertaining to online-based recruitment strategies for older adults.

**Objective:**

This study aimed to describe the lessons learned from the authors’ experience of recruiting a sample for PLAN (Preparing successful aging through dementia Literacy education And Navigation), an ongoing, community-based randomized controlled trial designed to promote the transition of community-dwelling Korean American older adults with probable dementia and their caregivers into the health care system. The authors also present online-based recruitment strategies focused on older adults reported in relevant published studies to compare with their experiences.

**Methods:**

Data sources included PLAN recruitment tracking files, study team meeting minutes, and interviews with community consultants. We also conducted a scoping review of published studies, searching PubMed in July 2021, and updated our search in September 2023. Eligibility criteria included (1) focus on older adults aged more than 65 years, (2) sample recruited from a community setting, and (3) inclusion and description of online-based recruitment strategies. Exclusion criteria (1) did not focus on adults older than 65 years in a community setting, (2) did not include or describe online-based recruitment strategies, or (3) used online-based methods but not for the purpose of recruitment. The review followed the PRISMA-ScR (Preferred Reporting Items for Systematic reviews and Meta-Analyses extension for Scoping Reviews). Information was extracted using a data charting table and synthesized by conducting a thematic analysis.

**Results:**

In total, 8 articles were included in the scoping review and primarily addressed health promotion and recruitment strategy evaluation. When compared with PLAN data sources, five key themes emerged as relevant to the online-based recruitment of community-dwelling older adults: (1) unfamiliarity with technology—limited digital literacy, (2) differences in internet access and use across older age groups, (3) providing technological support to promote recruitment, (4) successful and unsuccessful recruitment using social media, and (5) other diverse online-based methods of recruitment. In particular, direct quotes from multiple sources for the PLAN trial revealed technological challenges that were common among immigrant older adults as the study team used various online-based recruitment activities.

**Conclusions:**

The literature was limited in the discussion of online-based recruitment among older participants. Data sources revealed the digital divide and limited digital literacy, particularly among non–English-speaking immigrant older adults and their caregivers. The usefulness of online-based recruitment of older adults is uncertain due, in large part, to limited sociodemographic diversity noted in the samples recruited in the included studies. Future research should explore the role of race and ethnicity and other characteristics, such as socioeconomic status, sex, education, access to technology, and digital literacy, in relation to online-based recruitment for adequate representation of diverse older adults in research.

**Trial Registration:**

ClinicalTrials.gov NCT03909347; https://clinicaltrials.gov/study/NCT03909347

## Introduction

The US population is rapidly aging and expected to experience continued significant growth. According to the US Census Bureau, the population aged 65 years and more surged by 38% from 40.3 million in 2010 to 55.8 million in 2020, compared with a 7% increase in the total population [1]. More than 1 in 5 (22%) Americans will be aged 65 years and more by 2040, reaching 80.8 million in total [2]. The increasing proportion of older adults in the United States distinctly positions this group as a priority study population.

While American adults have seen overall gains in technology adoption and increased dependence on digital technologies, especially during the COVID-19 pandemic, the digital divide—the unequal access to digital technology and the internet—persists among older adults aged 65 years and older. For example, internet usage in 2021 was nearly ubiquitous among adults aged 18-64 years (18-29 years: 99%; 30-49 years: 98%; and 50-64 years: 96%) meanwhile internet usage trailed among older adults aged 65 years and older (75%) [3]. Nevertheless, the older adults’ group has also reported a steady increase in internet use over time, up from 14% in 2001, to 46% in 2011, to 75% in 2021 [3].

The COVID-19 pandemic presented unprecedented challenges to study teams with respect to the recruitment of study participants in community-based clinical trials. Globally, COVID-19–related restrictions were implemented including nationwide lockdowns and social distancing, and study teams had to quickly adjust study protocols to operate in an online-based environment. Engaging online with potential participants for recruitment activities (ie, identification, eligibility verification, informed consent, and enrollment) may be particularly challenging when the study population is older adults due to limited digital literacy [4]. Furthermore, limited digital access is also more common among older adults [5].

Despite rapid technological advancement and a considerably aging US population, there remains a gap in the literature pertaining to online-based recruitment strategies for older adults. Extant reviews related to online-based recruitment strategies included the examination of health research recruitment via Facebook (Meta) among adolescents [6], adults [7], and participants of all ages [8,9], as well as the examination of diverse digital tools for recruitment and retention of participants in randomized controlled trials (RCTs) [10], web-based mobile health studies [11], and recruitment performance of web-based respondent-driven sampling [12]. To our knowledge, a review specific to online-based recruitment methods among older adults has not been conducted.

PLAN (Preparing successful aging through dementia Literacy education And Navigation) is an ongoing, community-based RCT designed to promote the transition of community-dwelling Korean American older adults with probable dementia and their caregivers into the health care system for adequate diagnostic follow-up and care. The original protocol was designed to include community-based in-person outreach as the main recruitment approach. The start of PLAN recruitment coincided with the national lockdown and in-person activity restrictions resulting from the COVID-19 pandemic. Consequently, our study team had to devise online-based strategies to recruit study participants. The purpose of this paper is to describe our lessons learned from the experience of recruiting a sample comprised non–English-speaking older individuals who are cognitively impaired along with their caregivers for the PLAN trial. We also present our findings from a scoping review examining online-based recruitment strategies focused on older adults to compare with our experiences.

## Methods

### Description of the PLAN Trial

The PLAN trial uses an RCT design (ClinicalTrials.gov NCT03909347). Details of the study design and methods are described elsewhere [13]. Briefly, the study intervention consists of 1-hour dementia literacy education followed by monthly phone counseling for 6 months, all delivered by trained community health workers (CHWs). The study is dyad-based and the inclusion criteria for Korean American older adults are (1) self-identified as first-generation Korean American in the New York-New Jersey or Greater Washington Metropolitan Areas, (2) aged more than 65 years, (3) has probable dementia (1+ on clinical dementia rating), (4) has a caregiver who lives in the same household or has at least weekly interaction, and (5) able to consent or has a proxy available for consent. The study inclusion criteria for caregivers are (1) older than 18 years, (2) able to read and speak Korean, (3) living in the same household with the Korean American older adult or has at least weekly interactions, and (4) written consent to participate in the study and permission granted to the study team to audit medical records for linkage to health care. Eligible dyads (ie, Korean American older adult scoring clinical dementia rating 1+ and caregiver) are asked to fill out the study questionnaire at baseline, 3 months, and 6 months.

The trial was launched in late February 2021, when the COVID-19 pandemic was rampant and in-person recruitment activities were not allowed. Therefore, potential participants were recruited through ethnic mass media advertisements such as newspapers, television, and radio. The study team also began to use other online-based recruitment methods including the web pages of the participating community centers and local ethnic commercial websites as well as digital social platforms including Facebook, Instagram (Meta), Twitter (rebranded to X), YouTube (Google), and KakaoTalk (Kakao Corporation), a mobile messaging app popular among Koreans. The study team continues to use such online-based recruitment strategies to date.

### Sources of Data and Study Selection

Data sources used to identify online-based recruitment strategies and challenges for the PLAN trial included study recruitment tracking files and study team meeting minutes from the period of February to August 2021 when PLAN recruitment activities were solely online-based due to restrictions resulting from the COVID-19 pandemic. Additional data sources included interviews that took place in August 2021 immediately following the recruitment restriction period; interviewees included PLAN CHWs and community site coordinators as well as community consultants. We compared key lessons learned from the PLAN trial with a scoping review of published studies using digital technology to recruit community-dwelling older adults.

Our review followed the PRISMA-ScR (Preferred Reporting Items for Systematic reviews and Meta-Analyses extension for Scoping Reviews) checklist (Multimedia Appendix 1) [14]. The review protocol is not registered. In consultation with a health science librarian, we formulated a comprehensive search strategy comprising variations of the following search terms: “digital technology,” “recruitment,” “strategies,” and “older adults.” Additional restrictions applied included peer-reviewed literature published in English and with full-text availability. The detailed search strategy is presented in Multimedia Appendix 2. Our initial literature search involved the electronic database PubMed and was conducted on July 13, 2021, and updated on September 20, 2023.

The initial search yielded 505 nonduplicative records including titles and abstracts. Furthermore, 2 reviewers (among DM, J-YY, CP, and AJ) were assigned to screen each title and abstract independently for inclusion eligibility, and disagreements were resolved through consensus. Records were reviewed based on 3 eligibility criteria, that were (1) focus on older adults aged more than 65 years, (2) sample recruited from a community setting, and (3) inclusion and description of online-based recruitment strategies. Records were excluded if they (1) did not focus on older adults aged more than 65 years in a community setting, (2) did not include or describe online-based recruitment strategies, or (3) used online-based methods but not for the purpose of recruitment. After titles and abstracts were screened, a total of 103 full-text records were assessed for eligibility by 1 reviewer (DM) and a second reviewer (J-YY, CP, or AJ) reviewed every tenth record. In total, 6 studies met the eligibility criteria from the initial search. Using the same strategy, the second search yielded 91 additional titles and abstracts, with no duplicate records found. In addition, 2 reviewers (among DM, J-YY, and AJ) independently screened each title and abstract and 12 records entered the full-text review. One reviewer (DM) assessed each full-text record and a second reviewer (J-YY) reviewed every fourth record. With 2 records meeting the eligibility criteria in the updated search, a total of 8 records were included in this review. Articles were screened using Covidence (Veritas Health Innovation Ltd), a systematic review management platform.

### Data Extraction

Using a data charting table in Microsoft Excel, we systematically extracted the following information from each of the articles included in our review: first author’s last name, study design, study goal, country, sample characteristics (including sample size, key inclusion criteria, participant age cut-off or range, percentage of female participants, and percentage of White participants), recruitment methods along with associated yield rates, challenges related to technology and older adults, lessons learned, and identified themes.

### Data Analysis

We used a variety of methods to analyze relevant data from the PLAN trial and 8 relevant studies. Specifically, we used descriptive statistics, including frequencies and percentages, to calculate recruitment yield rates by different recruitment sources for the PLAN trial. For text-based data derived from the PLAN study team’s meeting minutes, stakeholder interviews, and recruitment-related information extracted from the 8 studies, we conducted a thematic analysis [15] to identify common themes related to recruiting older adults using online-based methods. To do this, the data were systematically coded to identify themes, which were then examined to identify those that were most common and relevant. The coding process informed the timing of interview completion, as we assessed data saturation by noting when no new themes emerged from the analysis.

Methodological rigor was ensured through the following approaches. First, for interview data collected in Korean from PLAN CHWs, community site coordinators, and Korean American community consultants, only the final results—themes, subthemes, and selected quotes—were translated and presented in English. This strategy minimized methodological challenges associated with repeated translations. Second, trustworthiness was established through several measures: (1) credibility was maximized by leveraging the bilingual research team’s extensive experience with the Korean American population and involving community consultants who are trusted members of the Korean American community; (2) dependability was enhanced through the use of multiple data sources and methods, ensuring triangulation of findings; (3) transferability was achieved by including verbatim transcripts and relevant quotes, enabling readers to assess the applicability of the study findings beyond the current context; and (4) confirmability was strengthened by revisiting the data through collaborative review by the initial coders and coauthors [16].

### Ethical Considerations

The Johns Hopkins Medicine Institutional Review Board reviewed and approved procedures of the human subjects research component of this study (IRB00242241). Written consent was not deemed as required by the institutional review board. Study staff members assured anonymity and confidentiality of participant information and responses and participants were not compensated for their participation. For the scoping review, ethics approval was not required as published articles were used.

## Results

### Overview

**Figure 1:**
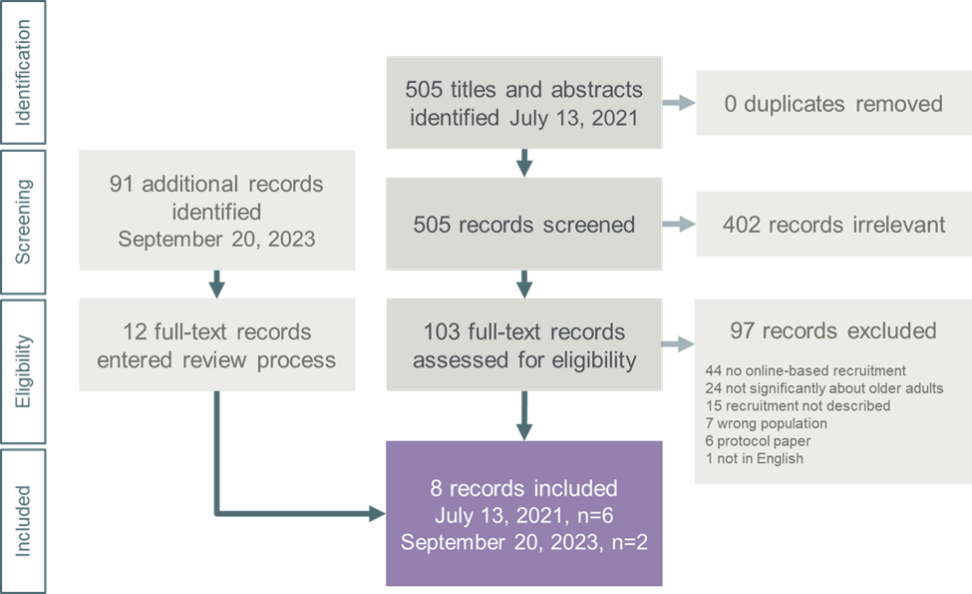
PRISMA-ScR (Preferred Reporting Items for Systematic reviews and Meta-Analyses extension for Scoping Reviews) flow diagram.

The initial and updated searches yielded 505 and 91 nonduplicative records, respectively, including titles and abstracts. Of these, 103 and 12 records from the initial and updated searches met the inclusion criteria for full-text review. A combined total of 8 records (6 from initial search and 2 from updated search) were included in this review (Figure 1).

### Characteristics of PLAN and Other Relevant Studies

Table 1 details key characteristics of the PLAN trial [13] and 8 relevant published studies [17-24] that used online-based strategies to recruit community-dwelling older adults. The scope of the relevant studies addressed health promotion and recruitment strategy evaluation. Of the 8 relevant studies, there were 3 observational studies [17,18,23], 2 secondary analyses [19,20], 2 RCTs [21,24], and 1 feasibility study [22]; 3 studies were conducted in the United States [18-20], while 2 studies were based in Australia [17,21], 2 studies based in Sweden [23,24], and 1 study based in Israel [22]. The 3 US-based studies involved English-speaking older adults with varying characteristics including 171 former caregivers of individuals with dementia (155/171, 91% White and 156/171, 91% female) [19]; 45 healthy older adults (90% White and 29/45, 64% female) [18]; and 795 adults, comprising 176 (22%) high-risk (having ischemic heart disease or congestive heart failure) older adults, 521 (66%) healthy older adults, and 98 (12%) healthy young adults (759/795 96% White and 504/795, 63% female) [20]. All 8 studies involved participants with normal cognition.

**Table 1:** Characteristics of studies using online-based recruitment strategies for older adults.

Reference	Study design; goal	Sample	Recruitment methods (% yield rate by source)	
			Online-based	Other
Han et al (PLAN^a^) [13]	RCT^b^ (2 arms); to determine the effect of a community-based, CHW^c^-delivered intervention for linkage to dementia medical services	United States; 461 Korean American adults; older adults aged more than 65 years with probable dementia (85%) and their caregivers aged more than 18 years (15%); sex not collected at intake	Contribution^d,e^: Facebook (0%), Instagram (0%), Twitter (0%), YouTube (0%), and KakaoTalk (1%); Korean community center e-newsletters (6%); Korean community websites (5%); local eHealth fair events and seminars (2%); and participation in local dementia caregiver online support groups (1%)	Contribution^d,e^: Adult day care centers (4%); senior centers (3%); churches (4%); newspaper (42%), radio (0%), local Korean television (1%); study flyers mailed to sites such as Korean markets, restaurants, clinics (9%); Meals on Wheels (2%); and word of mouth (20%)
Bajraktari et al [23]	Observational study; to evaluate the reach of a digital fall prevention intervention	Sweden; 173 Swedish older adults older than 70 years who previously experienced a fall or decline in balance in the past year; 70% (n=121) female	Contributions^f^: Local municipality Facebook page (11%) and local municipality homepage (1%)	Contribution^f^: Mailed brochure (47%); older adult meeting place coordinator (13%); newspaper, television, and radio (8%); family or acquaintance (5%); municipality staff (4%); senior center (2%); Friskis&Svettis (2%); patient associations (2%); and not specified (4%)
Bracken et al [17]	Observational study; to evaluate the cost and effectiveness of a range of promotional strategies used to recruit men to a large type 2 diabetes prevention trial	Australia; 1007 men aged 50-74 years with high-risk for developing diabetes; race or ethnicity breakdown not specified	Contribution^e,f^: Facebook page and advertising (2%, 2%); other internet (3 Google AdWords campaigns, study website, links on other websites; 1%, 2%); community promotions (4%, 5%); newspaper ads (0%, 0%); and nearby football club promotion (0%, 0%)	Contribution^e,f^: Mainstream media: radio (42%, 40%), television (20%, 22%), newspaper news (3%, 3%); mass mail-outs (17%, 17%); word of mouth (3%, 3%); health care provider (3%, 2%); radio news story (1%, 1%); and not specified (3%, 2%)
Cowie and Gurney [18]	Observational study; to demonstrate the effectiveness of using targeted Facebook advertising on Facebook to recruit for a Phase 1 clinical trial to assess safety, tolerability, and preliminary cognitive benefit of a compound being developed for the treatment of Alzheimer disease	United States; 45 healthy older adults aged 60-78 years; 64% (n=29) female; 90% White	Contribution^e^: Contract Research Organization website and intranet (10%) and Facebook advertising (73%)	Contribution^e^: Word of mouth, referral, and event (8%); print and newspaper ads (7%); and poster, flyer, direct mail, and billboard (4%)
Corey et al [19]	Secondary data analysis; to describe the use of internet-based recruitment in obtaining a sample inclusive of young and middle aged, young-old, and older-old former dementia caregivers	United States; 171 former dementia caregivers; 18-64 years (n=15, 9%), 65-74 years (n=51, 30%), more than 75 years (n=105, 61%); 91% (n=156) female; 91% (n=155) White	Contribution^f^: Facebook page (31%); caregiving-related websites (15%); and dementia-related websites (11%)	Contribution^f^: Professional referrals (42%) and other (1%)
Dill et al [20]	Secondary data analysis; to examine recruitment methods for a 5-year study comparing immune responses to an inactive influenza vaccine in older adults versus younger adults	United States; 795 adults; high-risk (having ischemic heart disease or congestive heart failure) older adults aged more than 60 years (n=176, 22%); healthy older adults aged more than 60 years (n=521, 66%); healthy young adults (n=98, 12%); 63% (n=504) female; 96% (n=759) White	Contribution^e^: Broadcast emails within research institution (4%)	Contribution^e^: Mailed letters (83%); physical referrals (5%); word of mouth and friend referrals (5%); and others (including newsletters, flyer, and seminars; 4%)
Miller et al [21]	RCT; to describe the recruitment strategies used and the success of each approach in recruiting older adults with type 2 diabetes into a 6-month community-based exercise and nutritional supplementation RCT	Australia; 198 adults aged 50-75 years with type 2 diabetes; 50-59 years (39%), 60-69 years (53%), older than 70 years (11%); 35% (n=70) female; race and ethnicity breakdown not specified	Contribution^e,f^: Facebook advertising (0%, 0%) and website advertising (8%, 10%)	Contribution^e,f^: Targeted mail-outs (41%, 39%); state-wide newspapers (28%, 27%); local newspapers (15%, 14%); physician referrals (2%, 5%); word of mouth (3%, 3%); flyers and community presentations (1%, 2%); radio advertising (1%, 0%); and not specified (0%, 0%)
Pettersson et al [24]	RCT; to describe recruitment strategies, reach, and participant characteristics for a digital fall-prevention intervention	Sweden; 1628 Swedish older adults aged more than 70 years who previously experienced a fall or decline in balance in the past year; 79% (n=1292) female	Contribution^f^: Social media including Facebook pages, paid Facebook advertisements, and Instagram (76%—breakdowns per platform not specified)	Contribution^f^: Newspaper advertisements (7%); family and friends (6%); senior citizen organizations (6%); articles in newspapers or radio (2%); other (2%); and not specified (1%)
Schwartz et al [22]	Feasibility study; to promote physical activity among older adults through live, online group training sessions over 8 weeks among older adults	Israel; 31 Israeli older adults aged 68-76 years medically approved to participate in moderate physical activity by a physician; 65% (n=20) female	Contribution^e,f^: Posts in Facebook groups (100%, 100%)	—^g^

^a^PLAN: Preparing successful aging through dementia Literacy education And Navigation.

^b^RCT: randomized controlled trial.

^c^CHW: community health worker.

^d^Referral sources from February 2021 to August 2021 (restrictions for in-person activities due to the COVID-19 pandemic).

^e^Contribution defined as the percentage of all potential participant expressions of interest resulting from a particular recruitment method.

^f^Contribution defined as the percentage of all participant enrollments resulting from a particular recruitment method.

^g^Not applicable.

Table 1 also summarizes key recruitment methods used in each study along with yield rates, indicated as contributions, by source. Notably, 2 types of yield rates were found across PLAN and the 8 studies, that were (1) the percentage of all potential participant expressions of interest resulting from a particular recruitment method [13,17,18,20-22], and (2) the percentage of all participant enrollments resulting from a particular recruitment method [17,19,21-24]. Online-based recruitment methods for the PLAN trial included YouTube and social media platforms such as Facebook, Instagram, Twitter (rebranded as X), and KakaoTalk, of which only KakaoTalk served as a referral source for potential participants (3/461, 1%). Community-based online strategies were more successful to draw interest; e-newsletters and websites by Korean community organizations (26/261, 6% to 28/461, 6%), local eHealth fair events and seminars (11/461, 2%), and dementia caregiver online support groups (5/461, 1%). Though not online-based, ethnic newspaper advertising was most successful to draw interest (194/461, 42%), followed by word of mouth (90/461, 20%). For the 8 relevant studies, the most widely used (all but 1 study by Corey et al [19]) online-based recruitment method was the social media platform Facebook, with varying yield rates from 0% to 100%, used in various forms such as Facebook pages [17,19,23,24], Facebook advertising [17,18,21,24], and Facebook groups [22]. Additional online-based recruitment strategies included other internet (Google AdWords campaigns, study websites, links on other websites) [17,23], listserve broadcast emails [20,24], and advertising on other and related websites [17-19,21,24] with varying yield rates from 0% to 15%.

### Recruitment of Older Adults Using Online-Based Strategies

We identified several key themes and lessons learned relevant to the recruitment of community-dwelling older adults (Table 2). These included unfamiliarity with technology, differences in internet access and use across older age groups, providing technological support to promote recruitment, successful and unsuccessful recruitment using social media, and other diverse online-based methods of recruitment.

**Table 2:** Key themes for online-based recruitment of older adults.

Theme	Lessons learned
Unfamiliarity with technology—limited digital literacy	Some participants struggle to understand basic concepts such as an “app” or smartphone versus 2G phone (PLAN^a^) Korean American older adults may reject the use of new technology, such as Zoom, because they are not used to nor familiar with it (PLAN)
Differences in internet access and use across older age groups	Online-based recruitment strategies are useful across age groups (young, middle-aged, young-old, and older-old), but provide greater access to young and middle-aged adults than older adults [19] Older-old (75 years and older) participants recruited only through caregiving- and dementia-related websites and none through Facebook [19]
Providing technological support to promote recruitment	Triaging participant technological capability through study intake call (PLAN) Zoom downloading assistance and tutorial through 1:1 precall with CHW^b^ (PLAN) Providing technological support through 1:1 15-minute online-based introductory session with study team member [22]
Successful and unsuccessful recruitment using social media	Social media yielded no results (Facebook, Instagram, Twitter, and YouTube), with the exception of KakaoTalk (1%; PLAN) Facebook was primary social media platform used across studies and presented mixed results (PLAN) [17-19,21-23] Successful: Facebook groups (100%) [22], Facebook advertising (73%) [18], and Facebook page (31%) [19] Unsuccessful: Facebook advertising (0%) [21], Facebook unpaid page and paid advertising (2%) [17], and Facebook page (11%) [23]
Using other diverse online-based methods of recruitment	Community center e-newsletters, ethnic community websites, e-health fair events or seminar, dementia caregiver online support groups with modest success (PLAN) Emails more successful with younger individuals; physician-signed letters more successful with older adults [20] Survey invitation to participate and survey link posted on websites of organizations related to caregivers and dementia less successful than Facebook pages; professional referrals most successful [19]

^a^PLAN: Preparing successful aging through dementia Literacy education And Navigation.

^a^CHW: community health worker.

#### Unfamiliarity With Technology—Limited Digital Literacy

Unfamiliarity with technology and limited technological proficiency can be a barrier to online-based recruitment for older adults. In the PLAN trial, we saw this among Korean American older adult participants who had varying levels of familiarity and experiences with technology like Zoom (Zoom Communications). Our CHWs reported that some Korean American older adults, when asked if they had a smartphone, could not distinguish between a smartphone and a 2G phone. Similarly, many older adults had difficulty finding the Google Play or the Apple Store to download an app such as Zoom or were unfamiliar with the word “app” or “Zoom.” One CHW shared her experience about the level of ability to use technology of PLAN study participants:

When I sent a Zoom link to a participant, in most cases, I had to send the link via text message because most KA older adults use phones [as opposed to computers]. Also, for most of them who received the Zoom invitation via text message, it was their first time using Zoom. Only those who use computers were able to communicate via email.

Community consultants shared a broad spectrum of responses to online-based study participation and affirmed that differences in technology use and familiarity have influenced the decisions of Korean American older adults to participate. A community consultant shared:

There are people who want to try new things, and there are people who absolutely reject things that they are not used to. Most older adults [I have encountered] are like that; not limited to Zoom, but because they don’t want to do anything that they are not familiar with.

However, others had previous experience and familiarity with Zoom through personal tasks such as attending telehealth medical visits and church meetings. Such older adults expressed positivity toward participating in PLAN via Zoom, as noted by another community consultant:

I did [Zoom] with my doctor about two weeks ago. If Zoom is the only way [to get a doctor’s appointment], we would do it...

#### Differences in Internet Access and Use Across Older Age Groups

Online-based recruitment may present different challenges and opportunities for older adults across different age groups. While rates of internet use and experiences of different age cohorts of older adults often go unacknowledged in the literature, Corey et al [19] used a 4-step internet-based approach for the recruitment of previous caregivers of people with dementia to participate in an online-based survey. The approach included the steps, such as (1) creation of study Facebook page, study invitation, and survey link posted to Facebook page; (2) study invitation and survey link posted to caregiver- and dementia-related websites; (3) study invitation and survey link posted to Facebook pages of relevant groups; and (4) study invitation and survey link email to principal investigator’s professional contacts [19]. Rates of referral through online-based sources were found to be comparable between the older-old subsample (46.7%) and the young-old subsample (47%), suggesting that internet accessibility and use between young-old and older-old adults is similar [19]. Nevertheless, diverse use of several online-based recruitment strategies was least successful in the recruitment of older-old (aged 75 years and older), with this age cohort only being recruited directly from caregiving- and dementia-related websites and none through Facebook [19].

#### Providing Technological Support to Promote Recruitment

When the PLAN study team conducted solely online-based research activities, from February through August 2021, early implementation revealed limited use of Zoom and varying levels of familiarity with technology among Korean older adults. One of the most common problems encountered by participants was the downloading process of Zoom. Community feedback supported the utility of technological assistance including that of a community consultant:

I don’t know how to use [Zoom], but I can do it if someone explains it to me side by side. But I don’t know how to go in and navigate through Zoom.

Responding to community feedback, we integrated an additional component to the PLAN intake call to triage interested potential participants with respect to technological capacity by posing questions related to device (eg, laptop, desktop, or smartphone), WiFi access, previous Zoom experience, and availability of someone to assist with Zoom set up. Based on their responses, individuals were categorized as either green to indicate technological self-sufficiency, or yellow, red, or black to signify lower levels of technological capacity; individuals of the latter groups were scheduled for a 1:1 precall with a CHW for Zoom downloading assistance and a tutorial before the cognitive screening. Similarly, Schwartz et al [22] scheduled online-based introductory sessions through Zoom with each interested potential participant to promote recruitment and provide technological assistance. The 15-minute 1:1 sessions included a Zoom tutorial as well as a brief overview of the study goals and required equipment for participation.

#### Successful and Unsuccessful Recruitment Using Social Media

All but 2 studies included in the review used social media as one of several recruitment strategies. Among the studies that leveraged social media, Facebook was the primary platform with a mix of targeted, paid advertisements, and unpaid postings [17-19,21-23]. Overall, the success of social media as a recruitment tool for older adults was mixed. For example, in an exercise and nutrition RCT for diabetes control, Miller et al [21] used paid Facebook advertisements with targeted key words based on age and location to capture attention of potential participants. Despite 16,600 clicks, among the 1157 total expressions of interest across recruitment types, only 5 came from social media; among those expressions of interest, no study participants were deemed eligible [21]. Similarly, in a study on diabetes prevention, Bracken et al [17] used both paid Facebook advertisements as well as unpaid Facebook posts. Facebook recruitment yielded a small proportion of expressions of interest as well as participant enrollment (2% and 2%, respectively), slightly more than other internet (combination of Google AdWords campaigns, study website, and links on other websites; 1% and 2%, respectively); radio advertising accounted for the largest proportion of both expressions of interest in addition to participant enrollment (42% and 40%, respectively) in the trial [17]. In a digital fall prevention intervention, Bajraktari et al [23] used the Facebook page of a local municipality, which yielded relatively low enrollment of 11% (19/173) compared with a 47% (81/173) yield from brochures mailed to households with at least 1 community-dwelling person aged 70 years or older as identified by a register provided by the local municipality. Similarly, social media was not an effective means of recruitment for the PLAN trial; relative to other methods, only 1% (3/461) of those screened for the PLAN trial were recruited via Facebook, Instagram, Twitter, YouTube, or Kakao Channel (a feature of a popular messaging app among Koreans—KakaoTalk).

A few studies successfully used social media to identify and enroll older adult participants. For example, using Facebook groups as the primary means of recruitment, 1 study surpassed their goal of 30 participants for an online-based physical activity intervention (using Zoom) for older adults in Israel [22]. In addition, in a study leveraging only social media channels that were unpaid, 24% (12/51) of the study sample aged 65-75 years were recruited via Facebook, although 0% (N=0) among those aged 75 years or older [19]. Recruitment through Facebook (unpaid) consisted of a dedicated page, created and managed by the PI, and posts to Facebook pages of other stakeholder groups [19]. There were no other indications of what other applications or websites were used other than postings on Facebook pages. Another study focused specifically on the effectiveness of social media recruitment to an Alzheimer treatment clinical trial via Facebook, as the study was failing to achieve the recruitment targets from its first phase of traditional recruitment methods (eg, billboards, newspaper advertising, word of mouth, personal referrals, and direct mail) [18]. Compared with the 11-week period of traditional recruitment methods that resulted in 178 inquiries and 6 enrolled subjects, the 8-week period with Facebook advertising resulted in 691 inquiries and 39 enrolled subjects. In the study by Cowie and Gurney [18], older men had a slightly higher engagement rate with the Facebook ads compared with women. However, women were found to have a higher engagement rate with their ad appealing to altruism (the recruitment campaigns targeted “typical” recruits by highlighting financial incentives for participation and “altruistic” recruits by highlighting the need for help) [18].

#### Using Other Diverse Online-Based Methods of Recruitment

There were other, nonsocial media online-based methods used for the recruitment of older adults with varying ranges of success. In the PLAN trial, other online-based methods used included community center e-newsletters, pop-up advertisements on websites for community sites, and e-health fair events and seminars organized by the trial’s 2 partnering community sites, and dementia caregiver online support groups. These diverse online-based methods yielded 16% (73/461) of potential participants referred to the study. Nonetheless, such methods were ineffective relative to traditional ethnic newspaper recruitment through which we identified 42% (194/461) of potential participants. Dill et al [20] used emails to recruit adults aged 20-40 years and older adults aged more than 60 years to compare immune responses with an inactive influenza vaccine between the groups and found that emails were more effective among younger individuals (57/94, 61%) compared with older individuals (25/645, 4%) [20]. Finally, 1 sample of former dementia caregivers was divided into 3 age groups (young and middle-aged: 18-64 years; young-old: 65-74 years; older-old 75+ years) to examine variation in internet-based recruitment and retention [19]. Corey et al [19] used websites of relevant caregiver and dementia organizations (Family Caregiver Alliance and Alzheimer’s Association) and found that older-old adults were most frequently directed to the study survey through such relevant websites (3/15, 20% and 4/15, 27%, respectively) compared with the young and middle-aged and young-old groups.

## Discussion

### Principal Results

Knowledge about online-based recruitment strategies among older adults is limited. This study set out to describe lessons learned from a community-based dementia literacy education intervention in cognitively impaired non–English-speaking older adults and their caregivers, as well as to compare such experiences with what is presented in the literature with respect to recruitment strategies that are online-based and older adult–focused. We learned that the most widely used online-based recruitment method across studies was the social media platform Facebook, with yield rates spanning 0% to 100% [13,17,19,21,22]. Despite an ongoing increase of internet use among older adults [3], recruiting older adults using other online-based methods such as internet (Google AdWords campaigns, study websites, and links on other websites), listserve broadcast emails, and advertising on other and related websites resulted in little to modest success with yield rates ranging from 1% to 15% [17-22].

The digital divide—one of the most significant social determinants of health [25]—was a theme that was particularly relevant to Korean American older adults and their caregivers in the PLAN trial [13]. Through direct recruitment encounters with potential study participants, the CHWs in the trial noted a 2G phone (aka, a “flip phone”) as a barrier to working toward online-based eligibility screening. A recent report by the Pew Research Center [26] revealed that in 2021, 97% of Americans owned a cellphone of some kind; 85% owned a smartphone. According to the report, smartphone ownership varied by age (from 96% for those aged 18-29 years to 61% for people aged more than 65 years) and level of education (from 75% for people with high school or less to 93% for those with college education). The rate of smartphone ownership was similar across White, Black, and Hispanic Americans. With the advent of a digital era, smartphone ownership often equates to connectivity to the world and access to the internet [27]. While it is unclear how many immigrant older adults use smartphones, our finding suggests the importance of addressing the issue of limited access to adequate digital devices in this vulnerable group.

Limited digital literacy was also a main barrier frequently reported in our and other studies included in the review [13,22]. Digital literacy is defined as “the ability to use information and communication technologies to find, evaluate, create, and communicate information, requiring both cognitive and technical skills.” In the PLAN trial, our CHWs recognized the challenges associated with limited digital literacy among Korean American older adults who often lacked a basic understanding of key technologies to enable them to follow through instructions for Zoom-based recruitment procedures. In particular, our observation of many Korean American older adults rejecting to learn “new things” (such as Zoom) is similar to the finding reported in a study involving community-dwelling adults [28]. In the study, participants were asked to perform a short task on a tablet involving a brief search with the same number of steps using either a familiar or a less familiar app. The authors found that older participants who used unfamiliar technology felt older after using the technology. Given these findings, trials using online-based recruitment should consider technological support for older adults as part of the recruitment process. Indeed, technological support through diverse methods (eg, triaging potential participants based on technological capabilities or 1:1 precall for assistance) was used to promote recruitment in our and other studies [13,22]. In addition, in a recently published experimental study involving individuals aged 55 and older, a series of in-person workshops incorporating hands-on training activities using the participants’ select mobile device, tablet, or smartphone (eg, touch and manual dexterity for mobile use, varying forms of communication such as text, image, voice, video, engaging tasks such as sharing of pictures, video, or other info, and other autonomous activities such as messaging for e-learning) resulted in significant improvement in digital literacy at 1-month follow-up [29]. Taken together, these results highlight some of the promising avenues to promote inclusiveness among older adults. In addition, future study teams may refer to a number of national and state-based resources that have been established to address digital literacy [30-33].

The usefulness of internet-based recruitment methods varied significantly among different age cohorts of older adults. Nevertheless, diverse use of several internet-based recruitment strategies was least successful in the recruitment of older-old (aged 75 years and older), with this age cohort only being recruited directly from caregiving- and dementia-related websites, which suggests the need for supplementary recruitment strategies for this age demographic rather than relying solely on internet-based methods [19]. Also, none of the recruited older-old participants were directed to the survey from Facebook, which suggests purposeful internet use among this age demographic and that social media use in this age group may not be sufficient for the use of social media–based recruitment methods, suggesting that response bias may be a significant consideration when using the internet in studies including older adults [19].

There was limited sociodemographic diversity in older adult participants recruited through internet-based strategies [17-22]. For example, we found that participant demographics, especially sample composition in terms of race or ethnicity, were not always reported; when reported, it was mainly White samples than non-White samples [18-20]. Furthermore, older participants included in the studies using internet-based recruitment had a high level of education and middle-upper class socioeconomic background [22]. More research is needed to explore the possible relationship between race or ethnicity, education, socioeconomic status, and ability to access and use online-based resources. There is also a demonstrated need to address the feasibility of online-based recruitment and research among older adults from lower socioeconomic backgrounds [19,22].

Success with social media as a recruitment tool for older adults varied. Social media is internet-based digital technology that allows the sharing of information and ideas and includes platforms such as Facebook, YouTube, X (previously called Twitter), Instagram, or WhatsApp. Given its wide reach and efficiency, researchers often find social media as an attractive tool for recruitment. Nonetheless, a recent integrative review [34] of 96 studies on social media use for research participant recruitment revealed that with the exception of 1, all other studies included in the review exclusively involved younger populations (eg, teens, young adults, or middle-aged participants); none specifically targeted older individuals with limited English proficiency. One of the studies included in the current review was a diabetes prevention trial and used Facebook for recruitment [17]. Yet, it only focused on older men and the authors were unable to determine whether lack of engagement via online-based strategies was due to the content of the advertisements and posts or sex-related differences with respect to the social media habits of men over the age of 50 [17]. Given the small number of relevant studies using social media to recruit older adults, more research is necessary to understand the effects of age and sex in relation to the utility of social media platforms for recruitment.

### Limitations

Our study has several limitations that warrant consideration. First, due to the timing of our exclusively online-based recruitment (early phase of COVID-19 pandemic), most potential participants who approached the study team had access to the internet. Naturally, we did not find any theme addressing the gap in internet access, although it is important to acknowledge that nationally, the rate of internet access among older adults aged 65 years and older is 75% [3]. The role of social media in our study is also noteworthy. For the most part, earlier articles using social media platforms for recruitment often lacked detailed information compared with more recent articles, providing gaps in reported online-based recruitment methodology and outcome. Furthermore, the articles in our review lacked ethnically diverse samples, and most studies were predominantly female, White, and with higher education, presenting a limitation in terms of generalizability of our findings (eg, 1 study used a Facebook algorithm to target individuals in higher income brackets [18]). Our review included articles published in English only and resulted in a total of 8 studies. The inclusion criteria around language restriction may have limited both the diversity and total number of articles, potentially influencing the level of comprehensiveness of our findings. In addition, due to the time-sensitive nature of our study, we used 1 primary database and selected PubMed given its widespread use and comprehensive coverage. This decision was made to maximize our search results within the constraints of limited time and resources. However, we note this as a limitation as relying solely on PubMed may have excluded relevant studies from other databases. Finally, we acknowledge the possibility of recalling bias. To minimize this, we used data sources that were based on written documentation (ie, recruitment tracking files and study team meeting minutes); in addition, interviews with CHWs, community site coordinators, and community consultants were conducted in August 2021, immediately following the 6-month period from February to August 2021 when PLAN recruitment activities were solely online-based due to restrictions resulting from the COVID-19 pandemic.

### Conclusions

Recruiting older adults using online-based strategies had varying success with most studies included in the review reporting more challenges than successes. As our nation continues to age, it is also becoming increasingly diverse [35]. Limited internet access and digital devices were noted as some of the main reasons for unsuccessful recruitment in the published studies of older adults included in our review; the same issue was also observed in our ongoing trial. The persistence of an age-based digital divide in the post–COVID-19 pandemic warrants both careful planning of recruitment activities to ensure technological support during the pre-enrollment period and the analysis of relevant recruitment outcomes as part of the study design. Such endeavors may promote better representation of those in research who are particularly vulnerable to the digital divide such as the older old, the less educated, and non–English-speaking ethnic minorities.

## Appendix

Multimedia Appendix 1 PRISMA (Preferred Reporting Items for Systematic Reviews and Meta-Analyses) literature review checklist.

Multimedia Appendix 2 PubMed search strategy.

## Abbreviations

PLAN: Preparing successful aging through dementia Literacy education And Navigation
CHW: community health worker
PRISMA-ScR: Preferred Reporting Items for Systematic reviews and Meta-Analyses extension for Scoping Reviews
RCT: randomized controlled trial

## Data Availability

All data that support this study’s findings will be made available from the corresponding author upon reasonable request.
