# Supplementary material for: Online-Based Recruitment Methods for Community-Dwelling Older Adults: Scoping Review and Lessons Learned From the PLAN Trial
Source: J Med Internet Res. 2025 Feb 25;27:e55082. doi: 10.2196/55082 (PMC11897674; doi:10.2196/55082)
Supplement: Multimedia Appendix 2 [file jmir_v27i1e55082_app2.pdf]

Appendix 1. PubMed search strategy

Search Number	Query	Results
5	#1 AND #2 AND #3 AND #4	505
4	strateg*	1,248,814
3	"Aged"[Mesh] OR "older adult*" [tiab] OR elder* [tiab] OR senior* [tiab] OR geriatric* [tiab]	3,426,727
2	"Patient Selection"[Mesh] OR recruit* [tiab] OR enroll* [tiab]	832,372
1	"Digital Technology"[Mesh] OR "Telecommunications"[Mesh] OR "Internet"[Mesh] OR "digital technology" [tiab] OR internet [tiab] OR "web-based" [tiab] OR "smart phone*" [tiab] OR videoconferenc* [tiab] OR "social media" [tiab] OR texting [tiab] OR "text messag*" [tiab]	250,175
